# Atmospheric Anæsthesia

**Published:** 1886-03

**Authors:** W. H. Richards

**Affiliations:** Knoxville, Tenn.


ARTICLE IV.

### "ATMOSPHERIC ANÆSTHESIA."

WHEN DISCOVERED AND APPLIED BY THE UNDERSIGNED.

BY W. H. RICHARDS, D. D. S., KNOXVILLE, TENN.

My boyhood days were spent in Salem, Va., at a time when coal was not used as a fuel, and fires were, of course, made of wood—to kindle the same often requiring much blowing, if the wood were damp and tinder scarce, (which was often the case at the time in particular of which I write, 1865) made memorable to many, who, on account of the recent unpleasantness, had to be their own hewers of wood, and do their own blowing, or freeze. I never thought to ask, before it came *my* turn to *blow*, whether the task was accompanied with unpleasantness or dizziness. No doubt, "Cuffy," from long practice in the art, which could not be acquired without much suffering—for suffering it was to me—was not affected thereby to any great extent; but at times I would be compelled to stop blowing and lie upon the floor, until my head and brains would be capable of taking in my surroundings.

I thought much of the effect of that class of "blowing" as I grew up, and when I became acquainted with the effects of nitrous oxide, in 1873, I was impressed with the similarity of sensation, and made this test upon myself, in the presence of an M. D. friend, who took my temperature and pulse, which, after as many rapid respirations as I was capable of, showed slight degree in temperature, augmented pulsation, much languor and lassitude, a tingling sensation in the fingers, such as is experienced under gas, or when, as we say, our hand is asleep.

In addition, there was much perspiration. In fact. I was exhausted, and might, or would, have submitted to a small operation, while in the acme of languor, indifference, or analgesia, if you please.

It was in 1875, when a very delicate young lady called to have a tooth, which I had been unable to cure of periostitis, extracted. She was afraid of gas, and I did not urge the use of it, because of her condition, and advised the rapid respiration, which was adopted.

At her suggestion, I permitted her to carry the rapid respiration as far as possible, to satisfy her of the effects, promising not to remove the tooth until a second trial, if satisfactory. The result was convincing, and she readily consented, on recovery, to having the tooth out, " when I get that way again." I removed the tooth, a lateral incisor, filled the root, and replanted the same. I called to see her, at her residence, next morning ; found her pleased with the result, and was surprised to hear her say she was confident I had given her gas, in some way, and would not be convinced to the contrary, until she tried the experiment of rapid breathing alone.

My experience teaches me that this kind of anæsthesia is fitted to those who are in an exalted nervous condition, and the secret of the success depends upon the party keeping his mind upon agitating a great deal of atmosphere. If you can keep the mind upon anything but the tooth, it will do as well.—*Southern Dental Journal.*

